# Editorial: Model organisms in experimental pharmacology and drug discovery 2022

**DOI:** 10.3389/fphar.2023.1143934

**Published:** 2023-03-29

**Authors:** Firzan Nainu, Catarina Jota Baptista, Ana Faustino-Rocha, Paula Alexandra Oliveira

**Affiliations:** ^1^ Department of Pharmacy, Faculty of Pharmacy, Hasanuddin University, Makassar, Indonesia; ^2^ Department of Veterinary Sciences, University of Trás-os-Montes and Alto Douro (UTAD), Vila Real, Portugal; ^3^ Centre for the Research and Technology of Agro-Environmental and Biological Sciences (CITAB), University of Trás-os-Montes and Alto Douro (UTAD), Vila Real, Portugal; ^4^ Instituto de Biomedicina, Universidad de León, León, Spain; ^5^ Department of Zootechnics, School of Sciences and Technology, Évora, Portugal

**Keywords:** model organisms, human diseases, drug discovery, drug interaction, herbal medicines (HM), Caenorabditis *elegans*, mice, rats

Model organisms are non-human species that have long been recognized as one of the crucial pillars in research, particularly in the field of experimental pharmacology and drug discovery (Hau, 2008). These organisms are widely employed in preclinical settings, providing a convenient way to model human diseases, explore the prospective pharmacological and toxicological effects of drug candidates, and elucidate the uncharted mechanisms of their action (Jota Baptista et al., 2022). Hence, studies using model organisms are essential for achieving scientifically sound progress within the field.

The Research Topic titled “*Model Organisms in Experimental Pharmacology and Drug Discovery 2022*” was established to showcase recent studies that use model organisms with substantial impact on the field of experimental pharmacology and drug discovery. This Research Topic was led by three Research Topic editors and one Research Topic coordinator that managed the whole editorial process, with the help of the Frontiers editorial team, for the submitted papers. This Research Topic was open for submission from 23 March 2022 to 8 August 2022. Of eight submissions, a total of five original articles were successfully accepted, giving promising insights into the use of model organisms and the future direction of experimental pharmacology and drug discovery. Except for Suarez et al. all studies use rodents as model organisms in their studies, illustrating the popularity and continued use to these species in particular, the most used throughout history in the context of drug discovery (Jota Baptista et al., 2022). The target drugs covered by this Research Topic Research Topic could not be more diverse, from anthelmintics to antidepressants or antinociceptives, for instance. Undeniably, all the works provide an essential contribution to improving our knowledge regarding new molecules or drug interactions, highlighting the relevance of the use of model organisms.

In the first article, Suarez et al. demonstrated the use of *Caenorhabditis elegans*, a free-living nematode, as a promising model to examine the pharmacodynamics and drug-drug interactions between anthelmintics, using ivermectin (IVM) and eprinomectin (EPM) as examples. Although this nematode has long been used in the screening of novel anthelmintic candidates, it has not been fully explored for other purposes such as the investigation of anthelmintic dose-response-time and drug-drug interactions. To achieve their purpose, Suarez et al. observed the effects of IVM and EPM on the movement of *C. elegans* by using an infrared motility assay. They found that the drug effect was increased, according to the exposure time, and worms could not recuperate once paralyzed. They also reported that EPM was less effective compared to IVM; though both drugs exhibited neither synergistic nor antagonistic profiles once combined. Overall, this study implies the pharmacological significance of the exposure time of each drug, rather than the combination of both drugs, to the improvement of their anthelmintic effects.

In the second article, Wan et al. confirmed the prospective role of puerarin in the treatment of acute alcoholism-induced oliguria. Using an integrative approach *via* network pharmacology analysis combined with experimental confirmation, the authors identified potential key targets of puerarin against acute alcoholism-induced oliguria in rats. Following a successful effort to establish a rat model of acute alcoholism, the authors revealed the involvement of cAMP signalling pathway in the puerarin-mediated pharmacological attenuation of oliguria in rats with acute alcoholism. This study has verified not only the potential role of puerarin in the management of acute alcoholism-induced oliguria but also the power of network pharmacology in combination with experimental validation to serve as a comprehensive platform to discover potent drug candidates with less adverse effects to treat human diseases.

In the third article, Lu et al. carried out a series of *in vitro* and *in vivo* experiments to demonstrate the anti-depressant-like effect of echinacoside (ECH). Using a combination of LPS-induced N9 microglial cells as the *in vitro* platform and mice with chronic unpredictable mild stress (CUMS) depression signatures as the *in vivo* model, the authors revealed that ECH could alleviate neuroinflammation-mediated depressive symptoms. Treatment of LPS-induced N9 microglial cells with ECH can reduce the production of NO without any adverse effects on cell viability and can suppress the activation of microglial cells, thus alleviating neuroinflammation. The authors also demonstrated that ECH can downregulate the expression of pro-inflammatory cytokines, such as IL-1β and TNF-α, and slightly upregulated the anti-inflammatory factors of IL-4 and IL-10, supporting the hypothesis that ECH may promote the microglial polarization from M1 to M2 with anti-inflammatory phenotypes. Further, the authors observed that ECH treatment can reverse CUMS-reduced sucrose preference in mice, probably due to suppression of the hippocampal neuronal damage, increased levels of 5-hydroxytryptamine, norepinephrine, and dopamine, and reduced expression of pro-inflammatory cytokines. Thus, their results demonstrated that ECH can alleviate depression-like behaviours, most likely by inhibiting neuroinflammation.

In the fourth article, Crawford et al. investigated the prospective antinociception and anti-allodynia effect of decursinol, the metabolite of its pyranocoumarins contained in the Angelica gigas Nakai (AGN) root alcoholic extract. To do so, the authors used two models of pain in mice: acute model of pain and model of chronic neuropathic pain. The results revealed that decursinol was able to elicit dose-dependent antinociception effect in mice experiencing acute thermal pain and anti-allodynia effect in the cisplatin-induced neuropathic mice. Although decursinol demonstrates promising pharmacological effects, the authors observed the development of tolerance to its pain-relieving properties. They found that full tolerance was detectable to both antinociceptive and anti-allodynia effects of decursinol after prolonged use. Therefore, decursinol demonstrates a promising use in the management of acute and chronic pain. Nevertheless, the authors also raise awareness for its rational use and future applications.

In the fifth article, Bervinova et al. studied the activity of the *Ficus tikoua* Bur. extract in the ethylene glycol-induced urolithiasis model of Sprague-Dawley (SD) rats. The authors used several parameters such as pathological signatures, biochemical and hematological parameters to evaluate the efficacy of treatment on the development of inflammation and urolithiasis. They found that the administration of *F. tikoua* Bur. extract led to the improvement of diuresis and the reduction of inorganic phosphates concentration as early as 6 weeks after treatment, comparable to Cystone^®^, a drug currently used to manage urolithiasis. However, different from Cystone^®^, treatment of the urolithiasis model of SD rats with *F. tikoua* Bur. extract can restore the level of lymphocytes, sodium, chlorine, and inorganic phosphates in the blood, comparable to the ones shown in the healthy control group. These results suggest that *F. tikoua* Bur. extract is a promising candidate for pharmacological treatment of urolithiasis with *F. tikoua* Bur. extract can restore lymphocytes, sodium, chloride, and inorganic phosphates to comparable healthy control group levels in the blood.

In conclusion, this Research Topic has provided recent updates and important findings related to the application of model organisms in experimental pharmacology and drug discovery. Continuous evaluations of reported methods and results are critically required to improve the planforms used in the discovery of new drugs with novel mechanisms of action and less toxicity that will offer better results in the disease management.
